# A Practical Quality Control Method for Saponins Without UV Absorption by UPLC-QDA

**DOI:** 10.3389/fphar.2018.01377

**Published:** 2018-12-11

**Authors:** Manjia Zhao, Yuntao Dai, Qi Li, Pengyue Li, Xue-Mei Qin, Shilin Chen

**Affiliations:** ^1^Institute of Chinese Materia Medica, China Academy of Chinese Medical Sciences, Beijing, China; ^2^Modern Research Center for Traditional Chinese Medicine, Shanxi University, Taiyuan, China

**Keywords:** saponins, *Astragalus membranaceus*, astragaloside IV, fingerprint, UPLC-QDA

## Abstract

Saponins are a class of important active ingredients. Analysis of saponin-containing herbal medicines is a major challenge for the quality control of medicinal herbs in companies. Taking the medicine Astragali radix (AR) as an example, it has been shown that the existing evaporative light scattering detection (ELSD) methods of astragaloside IV (AG IV) has the disadvantages of time-consuming sample preparation and low sensitivity. The universality of ELSD results in an inapplicable fingerprint with huge signals from primary compounds and smaller signals from saponins. The purpose of this study was to provide a practical and comprehensive method for the quality control of the astragalosides in AR. A simple sample preparation method with sonication extraction and ammonia hydrolyzation was established, which shortens the preparation time from around 2 days to less than 2 h. A UPLC-QDA method with the SIM mode was established for the quantification of AG IV in AR. Methanol extract was subjected to UPLC-QDA for fingerprinting analysis, and the common peaks were assigned simultaneously with the QDA. The results showed that with the newly established method, the preparation time for a set of samples was less than 90 min. The fingerprints can simultaneously detect both saponins and flavonoids in AR. This simple, rapid, and comprehensive UPLC-QDA method is suitable for quality assessment of RA and its products in companies, and also provides references for the quality control of other saponin ingredients without UV absorption.

## Introduction

Saponins are of great value in the development of new drugs or functional foods because of their wide distribution and various activities (Monschein et al., 2013). Some commonly used Chinese medicines, including ginseng (*Panax ginseng* C.A. Mey), Notoginseng (*Panax notoginseng*), Astragali radix (*Astragalus membranaceus*), licorice (*Glycyrrhiza*), dioscoreae rhizoma (*Dioscorea opposita* Thunb.), Ophiopogonis radix (*Ophiopogon japonicus*), all contain saponins (Ministry of Public Health of the People’s Republic of China, 2015). Therefore, the establishment of a simple and comprehensive quality control method is important for ensuring the quality of products containing saponins.

Because of the complexity of botanical ingredients, quantitative determination of index compounds (or active compounds) and the holistic analysis of fingerprints are widely used for the quality control of herbal medicines (Liu et al., 2007). However, saponins do not produce UV absorption or have terminal absorption. The ultraviolet detection method is used to detect the ultraviolet absorption peak of the compounds, with 203 nm often used as the detection wavelength for saponins (Qi et al., 2006). However, this method has weak sensitivity and low accuracy, and therefore, its usage rate gradually reduced. The existing evaporative light scattering detection (ELSD) method is currently used more as a general-purpose detector for saponins. Although the compounds with no UV absorption have relatively high sensitivity compared with the former, there are still some disadvantages such as insufficient sensitivity (Qi et al., 2008). The high-performance liquid chromatography with mass spectrometry (HPLC-MS) method for the determination of astragalosides has better selectivity and higher sensitivity, but it is relatively expensive and cannot be widely applied. QDA is a modular single quadruple mass detector. It is a small and inexpensive mass spectrometer detector compared with QTOF and a detector with high sensitivity for saponins compared with ELSD (Veryser et al., 2015; Yao et al., 2016). In this study, QDA was used to establish fingerprints and for the quantitative determination of astragalosides.

Astragalosides have important pharmacological functions in Astragali radix (AR) (Ministry of Public Health of the People’s Republic of China, 2015), which is one of the best known and widely used herbal medicines. It has been used over 2000 years for its immunomodulating (Huang et al., 2007; Zhang et al., 2009), for antioxidative (Sheih et al., 2011), and for antiinflammatory (Shon and Nam, 2003; Hoo et al., 2010). At present, quality control methods for AR include the determination of astragaloside IV with HPLC-ELSD in the Chinese Pharmacopoeia (Ministry of Public Health of the People’s Republic of China, 2015). In this method, sample preparation involves reflux extraction and liquid–liquid separation with *n*-butanol, which may take more than 2 days per sample. In addition, attempts were made to establish fingerprints for saponins by HPLC-ELSD for the overall quality control of AR (Ministry of Public Health of the People’s Republic of China, 2015). However, the fingerprint of saponins was overwhelmed by very large peaks from primary components, and the peaks for the saponins were too small because of the universality of the HPLC-ELSD method and the high proportion of primary components. For these reasons, quality control of saponins is time-consuming and lacks specificity or integrity. Hence, a simple, economical, and valid quality control method for AR is urgently required.

Taking AR as an example, the purpose of this study was to establish a simple and integrated quality control method for saponins in order to meet the requirements of product quality supervision during production. The astragaloside content was determined by the SIM mode of ultra-performance liquid chromatography (UPLC-QDA), and the full scanning mode was used to establish the fingerprint of the astragalosides and the main flavonoids in AR. It is a simple, fast, and holistic quality control method for saponins from AR.

## Materials and Methods

### Plants and Chemicals

Commercial samples of AR were collected from different places in China and authenticated as the dry roots of *Astragalus membranaceus* (Fisch.) Bge. var. *mongholicus* (Bge.) Hsiao using DNA barcoding method. The mean content of AG IV in all samples met the requirements of the Chinese Pharmacopoeia (Ministry of Public Health of the People’s Republic of China, 2015). A voucher specimen was deposited in the herbarium of the Institute of Chinese Materia Medica, China Academy of Chinese Medical Sciences.

**FIGURE 1:**
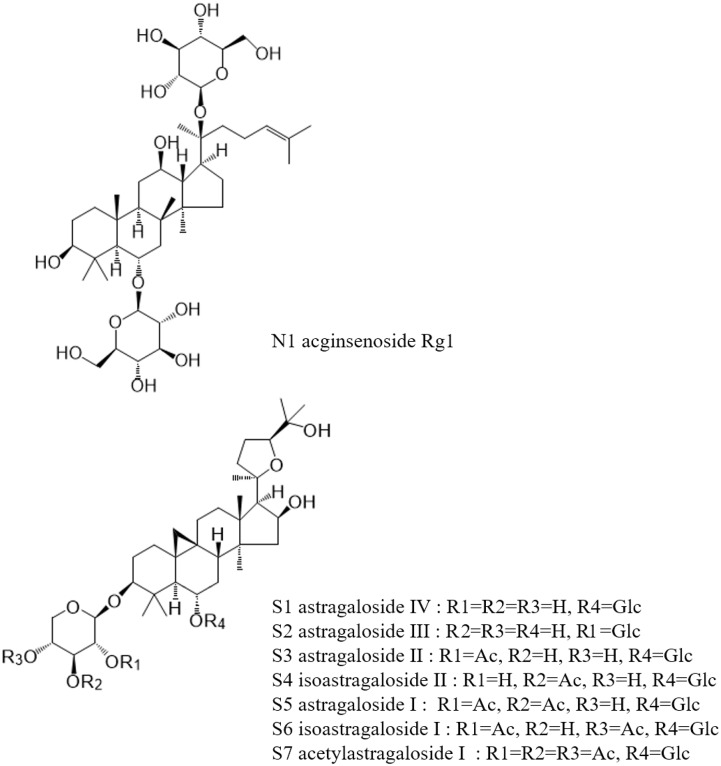
Chemical structure of marker constituents and the internal standard.

Saponin reference compounds, including astragaloside IV (AG IV, S1), astragaloside III (AG III, S2), astragaloside II (AG II, S3), astragaloside I (AG I, S5), and isoastragaloside I (iAG I, S6), and the internal standard ginsenoside Rg1 (N1), were obtained from the National Institute for the Control of Pharmaceutical and Biological Products. Their purities, as determined by HPLC, were above 98%. The structures of these compounds are shown in Figure 1. HPLC-grade acetonitrile (Fisher Scientific, United States), Optima LC-MS grade formic acid (Fisher Scientific, Czechia), and pure water (Wahaha, China) were used in the mobile phase. Other reagents and chemicals were of analytical grade. All solvents and samples were filtered through 0.22 μm membrane filters (Jinteng, Tianjin, China) before injecting into the HPLC.

### Astragaloside IV (AG) Analysis

#### Reference Solution Preparation

Five saponin reference compounds and the internal standard ginsenoside Rg1 were accurately weighed and formulated into standard solutions of 1 mg/mL with methanol and stored at 4°C for further use.

#### Sample Preparation

The dried roots of AR were milled to a homogeneous powder, and then sieved through a No. 65 mesh. Each powder sample, accurately weighed (2 g), was placed in a 50 mL centrifuge tube and ultrasonicated (40 kHz, 500 W) with 30 mL methanol for 30 min. After being centrifuged (about 3000 × *g*) for 5 min, the methanol solution was filtered. The residue was washed twice with 15 mL methanol, ultrasonicated (40 kHz, 500 W) for 5 min, centrifuged (about 3000 × *g*) for 5 min, and filtered. The filtrate was combined and evaporated with a rotary evaporator, and then the residue was redissolved in 10 mL of 10% (V/V) ammonia solution, shaken from time to time for 10 min, filtered through a membrane filter (0.22 μm), and then injected into the HPLC.

#### UPLC-QDA Conditions

Chromatographic analysis was performed on a Waters ACQUITY H-Class UPLC^®^ system, equipped with a quaternary solvent manager, sample manager, flow-through needle, high temperature column heater with active preheating, and QDA detector. Chromatographic separation was carried out at 35°C on a BEH Shield RP18 column (2.1 mm × 100 mm, 1.7 μm) (Waters). The mobile phase consisted of 0.1% formic acetonitrile (A) and 0.1% formic acid water (B), using an gradient elution of 5–30% A at 0–3 min, 30–40% A at 3–5 min, 40–100% A at 5–15 min, and 100–5% A at 15–18 min. The sample volume injected was 2.5 μL, and the flow rate was 0.4 mL/min.

The conditions of the electrospray ionization (ESI) source were as follows: ESI in positive mode; capillary voltage, 800 V; fragmentor, 15 V; sampling frequency, 5 Hz; Probe temperature 500°C. Ginsenoside Rg1 was detected in SIM 823.48 Da [M+Na]^+^ mode at 0–5.5 min; AG IV and AG III were detected in SIM 808.00 Da [M+Na]^+^ mode at 5.5–8 min; AG II and iAG II were detected in SIM 849.50 Da [M+Na]^+^mode at 0–8 min; AG I was detected in SIM 869.50 Da [M+H]^+^ mode at 0–8 min; iAG I was detected in SIM 891.50 Da [M+Na]^+^ mode at 0–8 min.

#### HPLC-ELSD Conditions

Quantitative analysis was performed using a 1200 Series HPLC (Agilent)–ELSD (Alltech 2000 ES). A YMC-Triart C18 column (250 mm × 4.6 mm. D.S-5 μm) was used for the chromatographic separations. The mobile phase consisted of 0.1% formic acetonitrile (A) and 0.1% formic acid water (B), using a gradient elution of 5–10% A at 0–5 min, 10–32% A at 5–10 min, 32–45% A at 10–30 min, 45–95% A at 30–35 min, and 95–20% A at 35–40 min. The injection volume was 20 μL, and the flow rate was 1 mL/min. ELSD was performed with air as the carrier gas at a flow rate of 2.5 L/min, and the nebulizer temperature was set to 100°C.

### Method Validation

#### Calibration Curves, Limits of Detection (LOD) and Quantification (LOQ)

Methanol stock solution of AG IV was prepared and diluted to appropriate concentration ranges (0.008, 0.009, 0.01, 0.06, 0.08, and 0.09 mg/mL) for the construction of calibration curves. The calibration curve was constructed using relative peak area (analyte/internal standard; *Y* axis), and the concentration of the standard (μg/mL; *X* axis). The diluted solution of the reference compound was further diluted with methanol to a series of concentrations for the gain of LOD and LOQ. The LOD and LOQ under the present chromatographic conditions were determined at a signal-to-noise (S/N) ratio of 3 and 10, respectively.

#### Precision, Repeatability, Stability, and Accuracy

Intra-day variations for six successive injections within 1 day were chosen to determine the precision of the developed method. Inter-day variations for three consecutive days were chosen to determine the precision of the developed method. To confirm the repeatability, six different working solutions from the same sample were prepared and analyzed. The sample stability test was determined with one sample during 1 day at 0, 0.5, 1, 2, 4, 8, 16, and 24 h. Over this period, the solution was stored at room temperature.

A recovery test was used to evaluate the accuracy of this method. For this, 1 mL of the above-developed AG IV standard solution of 1 mg/mL was combined with 1 g of the sample, and the mix was extracted as described above in the “Sample Preparation” section. Recovery was determined by comparing the difference between the mass of AG IV of the mix (sample + standard) (M1) and the mass of AG IV in the 1-g sample alone (M2), divided by the mass of AG IV standard added (M3), as shown in Equation (1). Recovery (%) = [(M1–M2)/M3] × 100% (1).

### Fingerprint Analysis

#### Sample Preparation

Each powder sample, accurately weighed (2 g), was placed in a 50 mL centrifuge tube and ultra-sonicated (40 kHz, 500 W) with 30 mL methanol for 30 min. After being centrifuged (about 3000 × *g*) for 20 min, the methanol solution was filtered through a membrane filter (0.22 μm), and then injected into the HPLC.

#### UPLC-QDA Conditions

Chromatographic separation was carried out at 30°C on a Waters CORTECS T3 column (2.1 mm × 100 mm, 1.6 μm). The mobile phase consisted of 0.1% formic acetonitrile (A) and 0.1% formic acid water (B) using an elution gradient of 2–19% A at 0–2 min, 19–42% A at 2–11.5 min, 42–55% A at 11.5–15 min, 55–65% A at 15–16.5 min, 65–75% A at 16.5–18 min, 75–100% A at 18–22.5 min, and 100–2% A at 22.5–24 min. The sample volume injected was 3 μL, and the flow rate was 0.4 mL/min.

The conditions of the ESI source were as follows: ESI in positive mode; capillary voltage, 800 V; fragmentor, 20 V; sampling frequency, 10 Hz. The QDA analysis worked using full scan mode, and the mass range was set at m/z 450–1200.

#### Method Validation

Intraday variations for six times within 1 day were chosen to determine the precision of the developed method. To confirm the repeatability, six different working solutions prepared from the same sample were analyzed. The sample stability test was determined with one sample during 1 day. In this period, the solution was stored at room temperature. By using the software “Similarity Evaluation System for Chromatographic Fingerprint of TCM,” the “correlation coefficients” and the “relative retention time (RRT)” and “relative peak area (RPA)” of the “common peaks” were calculated. Then the correlation coefficients and the RSD% of the RRT and RPA of common peaks were used as evaluation criterion, which could semi-quantitatively express the chemical properties in the chromatographic profiles of samples.

## Results and Discussion

### Optimization of UPLC Systems

**FIGURE 2:**
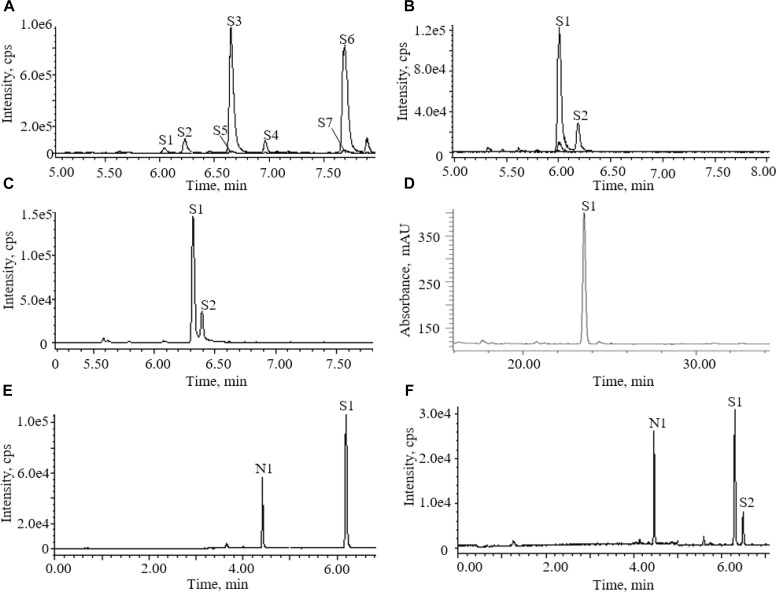
Chromatograms of astragalosides in different conditions: **(A)** UPLC-QDA chromatograms of astragalosides in different ion channels before addition of ammonia; **(B)** UPLC-QDA chromatograms of astragalosides in different ion channels after addition of ammonia; **(C)** UPLC-QDA chromatograms of Astragali radix (AR) extracts separated on CORTECS T3 column; **(D)** HPLC-ELSD chromatograms of AR extracts on YMC C18 column; **(E)** UPLC-QDA chromatograms of standard compounds; **(F)** UPLC-QDA chromatograms of AR samples. (S1) astragaloside IV; (S2) astragaloside III; (S3) astragaloside II; (S4) isoastragaloside II; (S5) astragaloside I; (S6) isoastragaloside I; (S7) acetylastragaloside I; (N1) ginsenoside Rg1.

Accord to literature, acetonitrile-water with 0.1% formic acid was used as mobile phase (Qi et al., 2009). Two columns were screened as fixed phase for the determination of AG IV in the AR extracts. Figure 2 shows the total ion chromatograms (TICs) of ESI (+) for the AR extracts separated on different columns. AG IV and AG III did not separate on a CORTECS T3 column, whereas good separation was achieved with a BEH Shield RP18 column (Figure 2).

An elution gradient was used for the determination of AG IV, instead of the isocratic elution methods used in Chinese Pharmacopoeia, and most literature (Ministry of Public Health of the People’s Republic of China, 2015). To avoid the interference of other compounds, the elution gradient was set to start with 5–30% of organic solvent for 3 min before the elution of target compounds. A UV spectrum showed that most of the flavonoids compounds were eluted out before the peak of AG IV. The elution gradient was optimized to ensure that the elution of most highly polar compounds took place before the elution of AG IV, avoiding the impact of other compounds on the determination of AG IV.

### Optimization of Sample Preparation

Sample preparation of AR in the determination methods of AG IV in the Chinese Pharmacopoeia includes 4-h solid-liquid extraction, liquid–liquid separation with butanol, taking more than 1 day, and column enrichment (Ministry of Public Health of the People’s Republic of China, 2015). One sample preparation will take more than 2 days, which is not suitable for monitoring a large number of products in a commercial situation. In this study, sonication extraction methods were used, instead of reflux extraction. The results showed that there was no statistical difference between the sonication and the reflux extraction methods (Supplementary Table S1).

After extraction, liquid–liquid separation with butanol and column enrichment were used to separate and enrich astragalosides from the extracts in the methods of Chinese Pharmacopoeia (Ministry of Public Health of the People’s Republic of China, 2015). This step was omitted in the sample preparation here and was done in the following UPLC analysis step, with a graduated wash starting with a high percentage of water elution, as described in the “Materials and Methods” section of this paper. This on-line elution with UPLC, instead of both solvent extraction and off-line column enrichment, saved a significant amount of time and also improved the sample preparation accuracy.

After extraction, ammonia solution was added into the extracts, and the amounts of ammonia compared. The results showed that the peak area of AG IV increased with the amount of ammonia solution, reaching its highest values with 10 mL or more of 10% (V/V) ammonia solution; therefore, 10 mL of 10% (V/V) ammonia solution was used in this study.

One important step for the sample preparation in Chinese Pharmacopoeia was reverse extraction with ammonia. It has proved that the purpose of this step was to transform other saponins into AG IV (Chu et al., 2014). The chromatograms of the astragalosides in different ion channels were recorded before and after the addition of aqueous ammonia treatment (Figure 2). They showed that the saponins detectable in the methanol extract of AR were AG IV (S1) and other astragalosides, including AG II (S3), iAG II (S4), AG I (S5), and iAG I (S6). After being processed with ammonia, AG II (S3), iAG II (S4), AG I (S5), and iAG I (S6) all disappeared and transformed into AG IV (S1) (Chu et al., 2014). The results indicated that other astragalosides, except AG III, could be converted into AG IV, and that the amount of AG IV detected was mainly the sum of AG I (S5), iAG I, AG II (S3), and AG IV (S1).

### Method Validation and Comparison With HPLC-ELSD

The calibration curve (*Y* = 29.215*X* + 0.2772) was successfully constructed using relative peak area for the *Y* axis and the concentration of standard as μg/mL for the *X* axis. The linearity of analytical response was acceptable with correlation coefficients higher than 0.99 offering a linear dynamic range of about two orders of magnitude. The LOD, LOQ, precision, stability, repeatability, and accuracy of the established methods for the determination of AG IV are summarized in Table 1 and Supplementary Table S2. All results of precision, stability, repeatability, and accuracy indicated that this method was valid.

**Table 1:** Comparisons the validation parameters of UPLC-QDA with HPLC-ELSD for analysis of astragaloside IV.

	UPLC-QDA	HPLC-ELSD
Retention time (min)	6.15	23.75
Linear	*Y* = 29.215*X* + 0.2772, *r* = 0.999	Ln(Y) = 1.535ln(X) + 0.7998, *r* = 0.998
Linearity range (mg/ml)	0.008–0.09	0.27–0.54
LOD (ng)	8	200
LOQ (ng)	25	500
Intra-day RSD (%) (*n* = 6)	1.96	0.74
Inter-day RSD (%) (*n* = 3)	1.77	0.21
Repeatability RSD (%) (*n* = 6)	2.77	1.56
Stability RSD (%) (*n* = 8)	2.10	1.40
Recovery mean (%)	100.95 (RSD 3.37%)	100.88 (RSD 3.19%)


The potential of UPLC-QDA was compared with the performance of HPLC-ELSD. Table 1 lists the performance index of UPLC-QDA compared with HPLC-ELSD for the analysis of AG IV. LOD and LOQ were seen to be low at 8 and 25 ng, compared with 200 and 500 ng for ELSD, which meant that the sensitivity was greatly improved by the use of UPLC-QDA instead of HPLC-ELSD. The higher sensitivity of UPLC-QDA than that of ELSD was also observed in sample detection. Figure 2 shows the chromatograms for the sample (d) with HPLC-ELSD and the chromatogram for standard compound (e) and sample (f) with UPLC-QDA. An obvious peak for AG III was observed with the established method, but it was not obvious in the chromatogram by HPLC-ELSD, which is attributed to the higher sensitivity of QDA than that of ELSD. The linearity range was also broadened with UPLC-QDA than ELSD and UPLC-QDA also showed a notably shortened analysis time. These advantages of UPLC-QDA indicate it successfully quantitative applications in quality analysis of RA. The precision, stability, and repeatability of the established UPLC-QDA method were not as good as the HPLC-ELSD method, but it is acceptable for the determination of AG IV in AR.

The established UPLC-QDA method was investigated for the analysis of AR. Fifteen samples from different batches were analyzed and the analytical contents were summarized in Table 2. All the analyses were carried out and repeated three times, and the data were recorded and expressed as the mean AG IV content. Table 2 shows the means for 15 batches detected with UPLC-QDA, and the results show a successful application of UPLC-QDA method to for the determination of AG IV in different AR sample.

**Table 2:** Quantitative analytical results of astragaloside IV in AR samples.

No.	Mean contents ± SD (%) (*n* = 3)
Sample_1	0.19 ± 0.0061
Sample_2	0.21 ± 0.0045
Sample_3	0.22 ± 0.0087
Sample_4	0.15 ± 0.0004
Sample_5	0.04 ± 0.0007
Sample_6	0.08 ± 0.0036
Sample_7	0.14 ± 0.0016
Sample_8	0.16 ± 0.0008
Sample_9	0.14 ± 0.0016
Sample_10	0.10 ± 0.0016
Sample_11	0.19 ± 0.0044
Sample_12	0.25 ± 0.0048
Sample_13	0.09 ± 0.0026
Sample_14	0.23 ± 0.0011
Sample_15	0.19 ± 0.0020


### Optimization of UPLC-QDA Conditions for Fingerprint

The elution gradient was optimized to achieve good separation for each peak in a short time. Several different gradients were tried and finally the gradient used in this study was selected, with good separation of each peak.

### Validation for Fingerprint Method

The correlation coefficients and the RSD% of the RRT and RPA of common peaks were calculated (Supplementary Tables S3–S5). The correlation coefficients of precision were higher than 0.989 and the RSD% of RPA was lower than 5.00. The correlation coefficients of the repeatability test were higher than 0.992 and the RSD% of RPA was lower than 8.00. The correlation coefficients of the stability test were higher than 0.984 and the RSD% of RPA was lower than 6.10, indicating that the sample remained stable for 1 day. All tests for precision, repeatability, and stability indicated that this method was valid and applicable.

### Establishment of Fingerprint of Saponins in AR

In this study, 15 samples were analyzed by the newly developed method. The mean chromatogram and correlation coefficients of the samples were calculated by using the similarity software, and it was found that correlation coefficients of AR samples were higher than 0.920, which indicated that all the samples tested have high consistency in quality.

**FIGURE 3:**
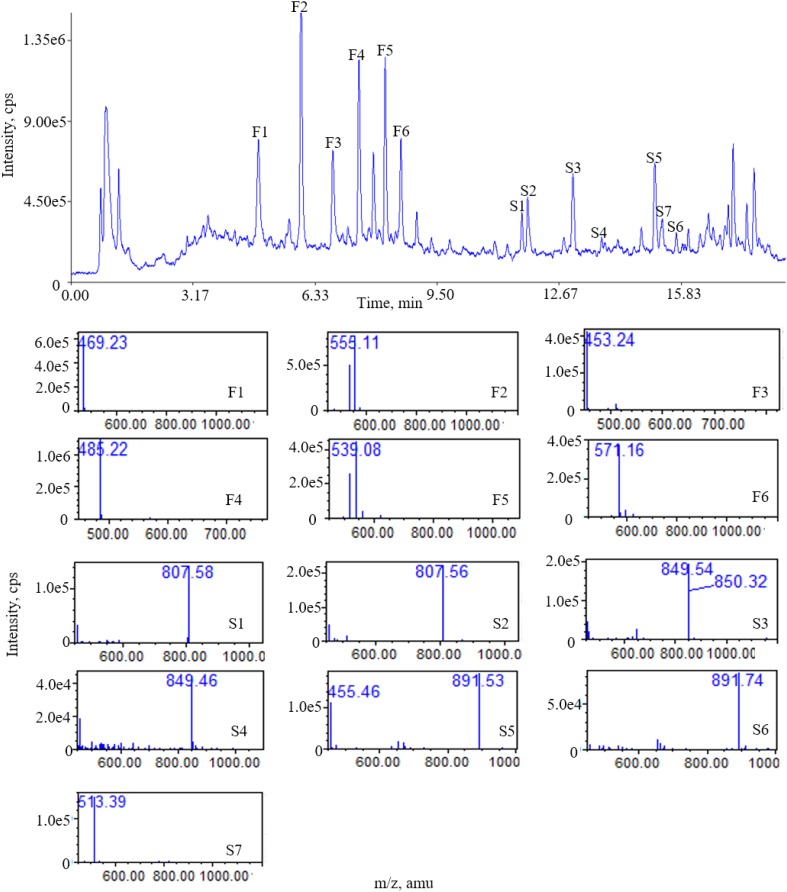
Total ion chromatogram (TIC) of ESI (+) QDA of saponins in AR samples: (F1) calycosin-7-O-β-D-glucoside; (F2) calycosin-7-O-β-D-glucoside-6^′′^-O-malonate; (F3) formononetin-7-O-β-D-glucoside; (F4) 9,10-dimethoxypterocarpan-3-O-β-D-glucoside; (F5) formononetin-7-O-β-D-glucoside-6^′′^-O-malonate; (F6) 3-hydroxy-9,10-dimethoxypterocarpan-7-O-β-D-glucoside-6^′′^-O-malonate; (S1) astragaloside IV; (S2) astragaloside III; (S3) astragaloside II; (S4) isoastragaloside II; (S5) astragaloside I; (S6) isoastragaloside I; (S7) cycloastragenol.

There were 13 “common peaks” existing in the chromatograms for the AR samples, which were assigned with ion mass analyzed with QDA and confirmed with reference compounds. Figure 3 shows the typical mass spectra for saponins. In the positive ion mode, six of the saponins generated typical [M+Na]^+^ ions, with mass accuracy at 807.5 + 42 n (*n* = 0 refers to AG IV, *n* = 1 refers to AG II/iAG II, *n* = 2 refers to AG I/iAG I). The six peaks of the flavonoids generated typical [M+Na]^+^ ions. The MS data for the six saponins and flavonoids in the positive ion mode are shown in Table 3. The RRT and RPA of the common peaks in the 15 samples were calculated and the data of the RPA was shown in Supplementary Table S6.

**Table 3:** On-line detected data for assigned compounds in Astragali Radix.

No.	Name	Observed RRT (min)	Relative peak area (RPA)	Observed m/z
F1	Calycosin-7-O-β-D-glucoside	0.85	0.38	469.23
F2	Calycosin-7-O-β-D-glucoside-6^′′^-O-malonate	1.00	1.00	555.11
F3	Formononetin-7-O-β-D-glucoside	1.12	0.18	453.24
F4	9,10-Dimethoxypterocarpan-3-O-β-D-glucoside	1.20	0.73	485.22
F5	Formononetin-7-O-β-D-glucoside-6^′′^-O-malonate	1.28	0.35	539.08
F6	3-Hydroxyl-9,10-dimethoxypterocarpan-7-O-β-D-glucoside-6^′′^-O-malonate	1.34	0.35	571.16
S1	Astragaloside IV	1.71	0.24	807.58
S2	Astragaloside III	1.74	1.47	807.56
S3	Astragaloside II	1.90	1.08	849.54
S4	Isoastragaloside II	2.02	0.25	849.46
S5	Astragaloside I	2.20	1.97	891.53
S6	Isoastragaloside I	2.28	0.09	891.74
S7	Cycloastragenol	2.26	0.02	513.39


The results shows that the fingerprint method established in this study can simultaneously detect a variety of saponins and flavonoids. In addition, the identities of each compound can be directly established by its mass number.

## Conclusion

A simple and fast quantification method for AG IV and an overall fingerprint of the main components (astragalosides and flavonoids) in AR have been established with UPLC-QDA. The established method was feasible for comprehensive quality evaluation of RA. The UPLC-QDA exhibits advantages over ELSD in sensitivity, peak assignment and simultaneous detections of components with and without UV absorption in fingerprint. The established methods provide references for the quality control of saponin ingredients without UV absorption. This study therefore provides suitable methods for the practical quality assessment of saponins in commercial situations.

## Author Contributions

YD, QL, X-MQ, and SC designed the study. MZ did the experiments. YD and MZ wrote the manuscript. All authors gave approval to the final version.

## Conflict of Interest Statement

The authors declare that the research was conducted in the absence of any commercial or financial relationships that could be construed as a potential conflict of interest.

## References

[B1] Chu C. Liu E. H. Qi L. W. Li P. (2014). Transformation of astragalosides from radix astragali under acidic, neutral, and alkaline extraction conditions monitored by LC-ESI-TOF/MS. *Chin. J. Nat. Med.* 12 314–320. 10.1016/s1875-5364(14)60062-5 24863360

[B2] Hoo R. L. Wong J. Y. Qiao C. Xu A. Xu H. Lam K. S. (2010). The effective fraction isolated from Radix Astragali alleviates glucose intolerance, insulin resistance and hypertriglyceridemia in db/db diabetic mice through its anti-inflammatory activity. *Nutr. Metab.* 7:67. 10.1186/1743-7075-7-67 20735814 PMC2936435

[B3] Huang G. C. Wu L. S. Chen L. G. Yang L. L. Wang C. C. (2007). Immuno-enhancement effects of Huang Qi Liu Yi Tang in a murine model of cyclophosphamide-induced leucopenia. *J. Ethnopharmacol.* 109 229–235. 10.1016/j.jep.2006.07.023 16963213

[B4] Liu A. H. Lin Y. H. Yang M. Guo H. Guan S. H. Sun J. H. (2007). Development of the fingerprints for the quality of the roots of Salvia miltiorrhiza and its related preparations by HPLC-DAD and LC-MS(n). *J. Chromatogr. B. Analyt. Technol. Biomed. Life Sci.* 846 32–41. 10.1016/j.jchromb.2006.08.002 16962399

[B5] Ministry of Public Health of the People’s Republic of China (2015). *Pharmacopoeia of the People’s Republic of China* Vol. 1 Beijing: Ministry of Public Health of the People’s Republic of China, 302–303.

[B6] Monschein M. Ardjomand-Woelkart K. Rieder J. Wolf I. Heydel B. Kunert O. (2013). Accelerated sample preparation and formation of astragaloside IV in Astragali Radix. *Pharm. Biol.* 10.3109/13880209.2013.839712 [Epub ahead of print]. 24171819

[B7] Qi L. W. Cao J. Li P. Wang Y. X. (2009). Rapid and sensitive quantitation of major constituents in Danggui Buxue tang by ultra-fast HPLC-TOF/MS. *J. Pharm. Biomed. Anal.* 49 502–507. 10.1016/j.jpba.2008.10.026 19056197

[B8] Qi L. W. Wen X. D. Cao J. Li C. Y. Li P. Yi L. (2008). Rapid and sensitive screening and characterization of phenolic acids, phthalides, saponins and isoflavonoids in Danggui Buxue Tang by rapid resolution liquid chromatography/diode-array detection coupled with time-of-flight mass spectrometry. *Rapid. Commun. Mass. Spectrom.* 22 2493–2509. 10.1002/rcm.3638 18651584

[B9] Qi L. W. Yu Q. T. Li P. Li S. L. Wang Y. X. Sheng L. H. (2006). Quality evaluation of Radix Astragali through a simultaneous determination of six major active isoflavonoids and four main saponins by high-performance liquid chromatography coupled with diode array and evaporative light scattering detectors. *J. Chromatogr. A.* 1134 162–169. 10.1016/j.chroma.2006.08.085 16982063

[B10] Sheih I. C. Fang T. J. Wu T. K. Chang C. H. Chen R. Y. (2011). Purification and properties of a novel phenolic antioxidant from Radix astragali fermented by *Aspergillus oryzae* M29. *J. Agric. Food Chem.* 59 6520–6525. 10.1021/jf2011547 21557623

[B11] Shon Y. H. Nam K. S. (2003). Protective effect of Astragali radix extract on interleukin 1beta-induced in fl ammation in human amnion. *Phytother. Res.* 17 1016–1020. 10.1002/ptr.1276 14595579

[B12] Veryser L. Taevernier L. Roche N. Blondeel P. De Spiegeleer B. (2015). Implementation of a single quad MS detector in high-throughput transdermal research of plant extracts. *J. Pharm. Biomed. Anal.* 115 594–602. 10.1016/j.jpba.2015.08.016 26320078

[B13] Yao C. L. Yang W. Si W. Pan H. Qiu S. Wu J. (2016). A strategy for establishment of practical identification methods for Chinese patent medicine from systematic multi-component characterization to selective ion monitoring of chemical markers: shuxiong tablet as a case study. *RSC Adv.* 6 65055–65066. 10.1039/c6ra10883k

[B14] Zhang R. P. Zhang X. P. Ruan Y. F. Ye S. Y. Zhao H. C. Cheng Q. H. (2009). Protective effect of Radix Astragali injection on immune organs of rats with obstructive jaundice and its mechanism. *World J. Gastroenterol.* 15 2862–2869. 19533807 10.3748/wjg.15.2862 PMC2699003

