# Supplementary material for: A Practical Quality Control Method for Saponins Without UV Absorption by UPLC-QDA
Source: Front Pharmacol. 2018 Dec 11;9:1377. doi: 10.3389/fphar.2018.01377 (PMC6298191; doi:10.3389/fphar.2018.01377)
Supplement: Supplementary file 1 [file Table_1.DOCX]

## supplementray materials

**Table S1**

Peak area comparison for ultrasound and reflux extraction methods (mAU).

| **extraction methods** | **Peak area average of astragaloside IV(n=3)** |
| --- | --- |
| Ultrasonic extraction | 249529 |
| Reflux extraction | 251641 |

**Table S2**

Recovery of astragaloside IV.

| **No.** | **M2 (mg)** | **M3 (mg)** | **M1 (mg)** | **Recovery (%)** | **Recovery mean (%)** | **RSD (%)** |
| --- | --- | --- | --- | --- | --- | --- |
| 1 | 1.44 | 1 | 2.43 | 99.42 | 100.95 | 3.37 |
| 2 |  |  | 2.48 | 103.60 |  |  |
| 3 |  |  | 2.49 | 104.68 |  |  |
| 4 |  |  | 2.39 | 95.10 |  |  |
| 5 |  |  | 2.45 | 101.27 |  |  |
| 6 |  |  | 2.46 | 101.62 |  |  |

**Table S3**

The correlation coefficients of precision.

| **No.** | **1** | **2** | **3** | **4** | **5** | **6** | **Mean chromatogram** |
| --- | --- | --- | --- | --- | --- | --- | --- |
| 1 | 1.000 | 0.991 | 0.992 | 0.980 | 0.986 | 0.989 | 0.996 |
| 2 | 0.991 | 1.000 | 0.990 | 0.981 | 0.979 | 0.982 | 0.994 |
| 3 | 0.992 | 0.990 | 1.000 | 0.980 | 0.986 | 0.989 | 0.996 |
| 4 | 0.980 | 0.981 | 0.980 | 1.000 | 0.973 | 0.978 | 0.989 |
| 5 | 0.986 | 0.979 | 0.986 | 0.973 | 1.000 | 0.981 | 0.991 |
| 6 | 0.989 | 0.982 | 0.989 | 0.978 | 0.981 | 1.000 | 0.993 |
| Mean chromatogram | 0.996 | 0.994 | 0.996 | 0.989 | 0.991 | 0.993 | 1.000 |

**Table S4**

The correlation coefficients of repeatability.

| **No.** | **1** | **2** | **3** | **4** | **5** | **6** | **Mean chromatogram** |
| --- | --- | --- | --- | --- | --- | --- | --- |
| 1 | 1.000 | 0.988 | 0.983 | 0.991 | 0.982 | 0.991 | 0.995 |
| 2 | 0.988 | 1.000 | 0.987 | 0.987 | 0.992 | 0.984 | 0.996 |
| 3 | 0.983 | 0.987 | 1.000 | 0.984 | 0.985 | 0.980 | 0.992 |
| 4 | 0.991 | 0.987 | 0.984 | 1.000 | 0.982 | 0.985 | 0.994 |
| 5 | 0.982 | 0.992 | 0.985 | 0.982 | 1.000 | 0.981 | 0.993 |
| 6 | 0.991 | 0.984 | 0.980 | 0.985 | 0.981 | 1.000 | 0.993 |
| Mean chromatogram | 0.995 | 0.996 | 0.992 | 0.994 | 0.993 | 0.993 | 1.000 |

**Table S5**

The correlation coefficients of stability.

| **Time** | **0h** | **1h** | **6h** | **10h** | **15h** | **24h** | **Mean chromatogram** |
| --- | --- | --- | --- | --- | --- | --- | --- |
| 0h | 1.000 | 0.984 | 0.986 | 0.977 | 0.987 | 0.981 | 0.993 |
| 1h | 0.984 | 1.000 | 0.991 | 0.971 | 0.991 | 0.986 | 0.995 |
| 6h | 0.986 | 0.991 | 1.000 | 0.979 | 0.995 | 0.987 | 0.997 |
| 10h | 0.977 | 0.971 | 0.979 | 1.000 | 0.974 | 0.972 | 0.984 |
| 15h | 0.987 | 0.991 | 0.995 | 0.974 | 1.000 | 0.984 | 0.996 |
| 24h | 0.981 | 0.986 | 0.987 | 0.972 | 0.984 | 1.000 | 0.992 |
| Mean chromatogram | 0.993 | 0.995 | 0.997 | 0.984 | 0.996 | 0.992 | 1.000 |

**Table S6**

Relative peak area of 13 common peaks in 15 samples.

| No. | Relative peak area | | | | | | | | | | | | | | |
| --- | --- | --- | --- | --- | --- | --- | --- | --- | --- | --- | --- | --- | --- | --- | --- |
|  | 1 | 2 | 3 | 4 | 5 | 6 | 7 | 8 | 9 | 10 | 11 | 12 | 13 | 14 | 15 |
| F1 | 0.24 | 0.20 | 0.26 | 0.32 | 0.28 | 0.77 | 0.65 | 0.57 | 0.46 | 0.37 | 0.39 | 0.40 | 0.24 | 0.28 | 0.31 |
| F2 | 1.00 | 1.00 | 1.00 | 1.00 | 1.00 | 1.00 | 1.00 | 1.00 | 1.00 | 1.00 | 1.00 | 1.00 | 1.00 | 1.00 | 1.00 |
| F3 | 0.11 | 0.11 | 0.07 | 0.11 | 0.18 | 0.27 | 0.23 | 0.30 | 0.21 | 0.16 | 0.12 | 0.20 | 0.17 | 0.27 | 0.16 |
| F4 | 0.38 | 0.42 | 0.41 | 0.48 | 0.76 | 1.40 | 1.29 | 1.06 | 0.90 | 1.16 | 0.67 | 0.70 | 0.55 | 0.45 | 0.39 |
| F5 | 0.49 | 0.45 | 0.50 | 0.28 | 0.31 | 0.25 | 0.34 | 0.32 | 0.33 | 0.16 | 0.27 | 0.35 | 0.40 | 0.42 | 0.40 |
| F6 | 0.31 | 0.30 | 0.31 | 0.29 | 0.41 | 0.35 | 0.34 | 0.39 | 0.44 | 0.46 | 0.32 | 0.43 | 0.23 | 0.47 | 0.27 |
| S1 | 0.01 | 0.02 | 0.03 | 0.00 | 0.01 | 0.00 | 0.04 | 0.00 | 0.00 | 0.00 | 0.00 | 0.03 | 0.00 | 0.08 | 0.02 |
| S2 | 0.06 | 0.08 | 0.12 | 0.03 | 0.04 | 0.16 | 0.13 | 0.09 | 0.06 | 0.07 | 0.25 | 0.13 | 0.05 | 0.10 | 0.07 |
| S3 | 0.05 | 0.09 | 0.10 | 0.04 | 0.02 | 0.13 | 0.13 | 0.07 | 0.04 | 0.04 | 0.07 | 0.12 | 0.03 | 0.08 | 0.07 |
| S4 | 0.00 | 0.00 | 0.00 | 0.00 | 0.01 | 0.00 | 0.06 | 0.05 | 0.00 | 0.00 | 0.03 | 0.00 | 0.01 | 0.03 | 0.06 |
| S5 | 0.08 | 0.14 | 0.16 | 0.09 | 0.04 | 0.30 | 0.20 | 0.19 | 0.09 | 0.06 | 0.15 | 0.15 | 0.08 | 0.13 | 0.12 |
| S7 | 0.08 | 0.00 | 0.00 | 0.00 | 0.00 | 0.00 | 0.00 | 0.00 | 0.00 | 0.02 | 0.00 | 0.02 | 0.00 | 0.10 | 0.01 |
| S6 | 0.00 | 0.00 | 0.00 | 0.00 | 0.00 | 0.00 | 0.01 | 0.03 | 0.01 | 0.01 | 0.00 | 0.03 | 0.00 | 0.00 | 0.00 |
